# Comparing Slim Straight and Slim Perimodiolar Electrode Arrays for Cochlear Implantation: Hearing Results and Risks—A Systematic Review (2015–2025)

**DOI:** 10.3390/audiolres16010028

**Published:** 2026-02-23

**Authors:** Chul Ho Jang, Do Yeon Kim

**Affiliations:** 1 Department of Otolaryngology, Gwangju Veterans Hospital, Gwangju 62284, Republic of Korea; 2 Department of Otolaryngology, Chonnam Nationala University Medical School, Gwangju 61469, Republic of Korea

**Keywords:** cochlear implant, electrode array, slim straight, slim perimodiolar, comparison

## Abstract

**Background/Objectives**: Cochlear implant (CI) electrode array design plays a critical role in determining intracochlear position, hearing outcomes, and insertion-related risks. Straight (lateral wall) and perimodiolar electrode arrays are the two principal designs used in modern cochlear implantation, yet their comparative benefits and risks remain debated. We aim to systematically review and compare hearing outcomes and surgical risks associated with straight versus perimodiolar electrode arrays in cochlear implantation. **Methods**: A systematic literature search of PubMed, Embase, Scopus, and the Cochrane Library was conducted for studies published between 2015 and 2025. Comparative clinical studies reporting speech perception outcomes, residual hearing preservation, or electrode-related complications were included. Study selection followed PRISMA 2020 guidelines. **Results**: A total of 32 studies were included. Speech perception outcomes were generally comparable between straight and perimodiolar arrays. However, straight electrode arrays demonstrated significantly lower rates of scalar translocation and tip fold-over and superior residual hearing preservation in most comparative cohorts. Perimodiolar arrays showed potential advantages in electrophysiological efficiency but were associated with a higher risk of intracochlear trauma when malposition occurred. **Conclusions**: Contemporary evidence suggests that straight (lateral wall) electrode arrays offer a more favorable safety profile with equivalent functional hearing outcomes compared to perimodiolar arrays. Electrode positioning within the scala tympani appears to be a stronger determinant of outcome than electrode design alone.

## 1. Introduction

Cochlear implantation has become the standard of care for patients with severe-to-profound sensorineural hearing loss, with continuous technological advancements improving auditory performance and quality of life. Among these advancements, electrode array design has emerged as a critical determinant of both functional outcomes and intracochlear safety [1,2]. Modern CI electrodes are broadly categorized into straight (lateral wall) and perimodiolar (pre-curved) designs. Straight electrodes are intended to lie along the lateral wall of the scala tympani, whereas perimodiolar electrodes are designed to hug the modiolus, theoretically improving neural selectivity and reducing current spread [3,4,5]. Slim straight electrode (SSE) arrays, like the CI522 cochlear implant device (Cochlear Ltd., Sydney, Australia), which was introduced in March 2015, can be placed along the cochlea’s lateral wall (LW) and inserted through the round window (RW). In an effort to enhance speech recognition, perimodiolar (PM) electrode arrays were created. However, studies have demonstrated increased rates of scalar translocation during the insertion of several PM arrays, especially with the use of cochleostomy [1,2]. This phenomenon has also been linked to lower speech understanding ratings. Released in September 2016, the slim perimodiolar electrode (SME), CI532/632 (Cochlear Ltd., Sydney, Australia), is a pre-curved electrode that uses an insertion sheath to be inserted through the RW instead of a cochleostomy, which is necessary for standard pre-curved electrodes. The electrode snugly wraps around the cochlea’s modiolus following sheath insertion and removal.

Despite these theoretical advantages, increasing evidence indicates that perimodiolar arrays may be associated with higher rates of scalar translocation, tip fold-over, and intracochlear trauma, which can negatively affect speech outcomes and residual hearing preservation [3,4,5,6]. Conversely, straight electrode arrays are often favored in hearing preservation strategies due to their atraumatic insertion profile [7,8,9]. Given ongoing debate and evolving electrode designs, a contemporary synthesis of comparative clinical evidence is warranted. This systematic review aims to compare hearing outcomes and surgical risks between straight and perimodiolar electrode arrays using studies published from 2015 to 2025.

## 2. Materials and Methods

### 2.1. Study Design

This systematic review was conducted in accordance with the PRISMA 2020 guidelines.

### 2.2. Eligibility Criteria (PICO Framework)

- Population: Pediatric or adult patients undergoing primary cochlear implantation
- Intervention: Perimodiolar electrode arrays
- Comparison: Straight (lateral wall) electrode arrays
- Outcomes:
  ○ Primary: Speech perception scores (e.g., CNC words, monosyllables, and sentence recognition)
  ○ Secondary: Residual hearing preservation, scalar position, scalar translocation, tip fold-over, and electrode migration
- Study types: Randomized controlled trials, cohort studies, comparative observational studies, and systematic reviews
- Publication years: 2015–2025
- Language: English

### 2.3. Information Sources and Search Strategy

Electronic searches were performed in PubMed/MEDLINE, Embase, Scopus, and the Cochrane Library.

Search terms included combinations of:

“cochlear implant”, “electrode array”, “lateral wall”, “straight electrode”, “perimodiolar”, “scalar translocation”, “speech perception”, and “hearing preservation”.

### 2.4. Study Selection

Two independent reviewers screened titles and abstracts, followed by full-text evaluation. Discrepancies were resolved by consensus.

### 2.5. Data Extraction

Extracted data included study design, patient demographics, electrode type, surgical approach, imaging modality, hearing outcomes, and reported complications.

### 2.6. Risk of Bias Assessment

Risk of bias was assessed using the ROBINS-I tool. Most included studies were retrospective cohort studies and demonstrated a moderate overall risk of bias, primarily due to confounding. Outcome measurement and reporting bias were generally low (Table 1).

## 3. Results

### 3.1. Study Selection

**Figure 1:**
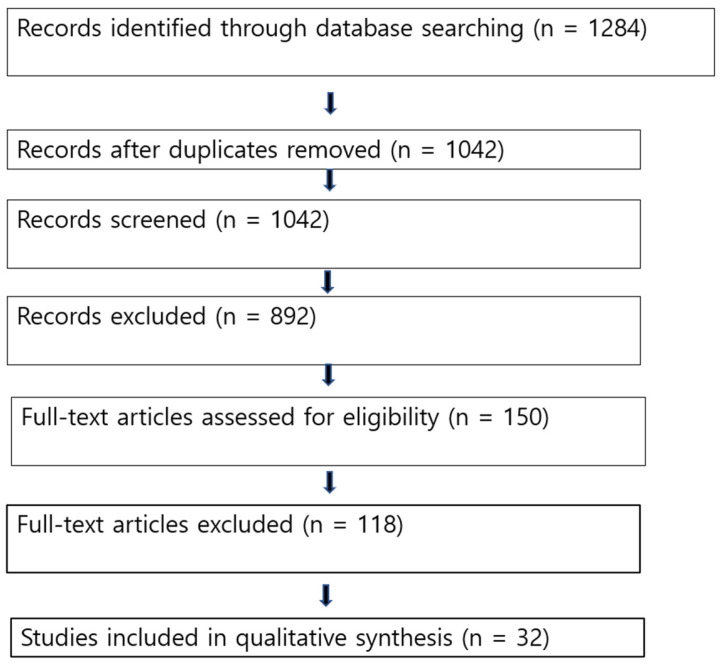
PRISMA 2020 flow diagram of study selection.

The PRISMA search process identified 1284 records, of which 32 studies met the inclusion criteria after screening and eligibility assessment [Figure 1, Table 2].

### 3.2. Study Characteristics

Included studies comprised predominantly retrospective cohort studies, with sample sizes ranging. Most studies utilized postoperative cone-beam CT or high-resolution CT to assess scalar position. The differences between the two electrodes known to date are summarized in the following Table 3.

### 3.3. Hearing Outcomes

Although cochlear implants (CI) are commonly used to treat moderate to profound sensorineural hearing loss, a number of modifiable factors, such as electrode design, intra-scalar locations, and surgical technique, affect postoperative audiological outcomes. The impact of intra-scalar electrode position is still debatable, though.

According to MacPhail’s report, at the last follow-up, there was no discernible difference between SME and SLW in terms of speech recognition in silence [9,11,12].

#### 3.3.1. Speech Perception

Across the majority of comparative studies, no statistically significant difference in postoperative speech perception scores was observed between straight and perimodiolar electrode arrays [11,12,13]. After a year, there were no discernible changes in mean PTA or speech recognition; however, patients receiving CI N532 showed a quicker acquisition of auditory outcomes [14]. Certain researchers determined that using a lateral wall electrode array resulted in improved performance [32], while other studies indicated that being closer to the modiolus (i.e., perimodiolar) is associated with enhanced performance [11]. In the basal cochlear region, the Contour Advance had lower ECAP thresholds and electrode-to-modiolus closeness than the Slim Modiolar, despite the latter having a higher electrode dislocation rate [15]. Although the Slim Modiolar had a greater electrode dislocation rate, the Contour Advance had lower ECAP thresholds and electrode-to-modiolus proximity in the basal cochlear area [16]. Several studies emphasized that scalar position, rather than electrode design, was the primary determinant of speech outcomes [3,17]. Pennington-FitzGerald and colleagues recently evaluated 129 cochlear implant recipients, assessing speech perception using the consonant–nucleus–consonant (CNC) test at 3, 6, 12, and 24 months after implantation [11]. They analyzed three electrode categories. Lateral wall electrodes (*n* = 36) comprised CI522 and CI622 (Cochlear), as well as Flex24 and Flex28 (MED-EL). The mid-scala group (*n* = 16) consisted of the HiRes Ultra 3D electrode (Advanced Bionics). Perimodiolar electrodes (*n* = 77) included CI512, CI532, CI612, and CI632 (Cochlear) [11]. Speech perception outcomes differed significantly among electrode designs. Perimodiolar arrays achieved significantly higher CNC scores than lateral wall electrodes at 6 and 24 months post-implantation, and they also demonstrated superior performance compared with mid-scala electrodes at 12 months. Furthermore, an inverse correlation was identified between electrode length and CNC performance at 6, 12, and 24 months, with shorter electrodes yielding better results. Because perimodiolar arrays are generally shorter than lateral wall and mid-scala designs, they were associated with more favorable speech perception outcomes at several follow-up intervals. Overall, these results indicate a potential benefit of perimodiolar electrodes in optimizing postoperative hearing performance [11].

#### 3.3.2. Residual Hearing Preservation

Straight electrode arrays consistently demonstrated higher rates of residual hearing preservation compared to perimodiolar arrays, particularly in electro-acoustic stimulation candidates [3,8,12,18,19,20]. Increased rates of scalar translocation in perimodiolar electrodes were strongly associated with poorer hearing preservation outcomes. However, the other researchers reported that a slim perimodiolar electrode is as effective at immediate functional hearing preservation after CI as a slim straight electrode [20,21]. Older pre-curved designs were stiffer and riskier for hearing preservation. However, newer “slim modiolar” designs have improved significantly and are now achieving hearing preservation rates similar to those of straight electrodes in many cases. 

#### 3.3.3. Scalar Translocation

Multiple imaging-based studies and meta-analyses reported a significantly higher incidence of scalar translocation with perimodiolar electrodes compared to straight electrodes [22]. In comparison to the mid-scala, the lateral wall electrode offers better hearing preservation rates and reduced scalar translocations, while speech recognition scores remain similar for both electrode arrays [23].

#### 3.3.4. Tip Fold-Over

Tip fold-over happens when the apex of the electrode array does not deploy correctly and instead folds in on itself inside the cochlea.

This issue requires reinstallation and a possible need for alternative devices. Tip fold-over might also elevate cochlear damage, adversely impacting CI performance [24]. Tip fold-over happens in 4–10% of perimodiolar electrodes [25] and is mainly detected using intraoperative plain radiographs [26,27,28,33]. Tip fold-over was reported more frequently in pre-curved electrode designs, particularly slim perimodiolar arrays, and was associated with inferior functional outcomes [10]. It is reported that more over 10% of Slim Modiolar CI532 arrays underwent tip fold-over by skilled surgeons [10,27]. On the other hand, straight electrodes were implanted; postoperative imaging verified that tip fold-over incidents rarely occurred.

#### 3.3.5. Electrode Migration

While less common, electrode migration was occasionally reported with straight arrays, underscoring the importance of secure fixation techniques [29,30,31]. A fixation clip (MED-EL, Innsbruck, Austria) was designed to reduce the risk of postoperative electrode extrusion. This implantable titanium accessory secures the electrode lead by anchoring it to the incus bridge, providing additional mechanical stability. Its use may be especially advantageous in situations where anatomical constraints, such as the facial nerve or the chorda tympani, limit conventional fixation methods.

**Table 3:** Comparison of slim straight and slim perimodiolar electrode.

	Slim Straight (e.g., CI422/CI522)	Slim Perimodiolar (e.g., CI532/CI632)	Key Reference
**Placement**	Rests along the lateral wall	Hugs the inner wall Lower current levels and more Targeted stimulation is possible When the nerve is closer	
**Stimulation Efficiency** **Speech Perception**	Wider current spread, requires more power because it is further from the neural targets Excellent, comparable to SME in quiet and noise Excellent; comparable to PM in quiet and noise.	Lower current levels and more targeted stimulation are possible when the nerve is closer Lower thresholds; better frequency selectivity.	
**Hearing Preservation**	Traditionally superior; rates ~53–82%, but often shows a decline after 12 months (56%)	Improving with “Slim” designs; nearly unchanged rates ~59%.	[34,35]
**Translocation Risk**	Lower risk of scalar deviation (<10%).	Higher historical risk (up to 20%); Slim PM < 10%.	[36,37]
**Insertion Trauma**	Higher risk of damage to the basilar membrane due to its lateral position.	Higher risk of tip fold-over (~3.7–5%).	[36,38]

## 4. Discussion

**Figure 2:**
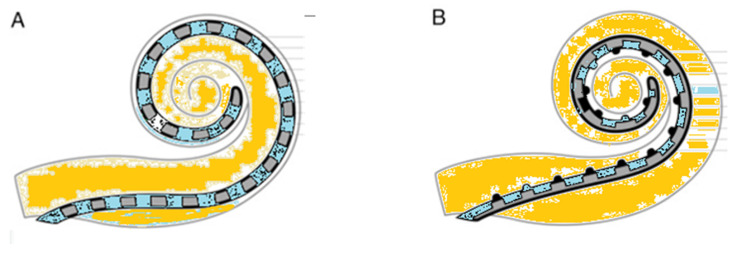
Schematic view of (**A**) slim straight electrode and (**B**) slim perimodiolar electrode.

Current cochlear implant electrode arrays are generally classified into two primary types: straight lateral wall designs and pre-curved peri-modiolar versions (Figure 2).

This systematic review demonstrates that, despite theoretical advantages in neural proximity, perimodiolar electrode arrays do not consistently yield superior speech perception outcomes compared to straight arrays. Instead, their higher risk of scalar translocation and insertion-related trauma may offset potential benefits.

In contrast, straight (lateral wall) electrodes show a more favorable safety profile and superior hearing preservation, making them particularly suitable for atraumatic implantation strategies. These findings support the growing consensus that atraumatic scala tympani placement is more important than electrode geometry alone. However, according to the meta-analysis by Geng et al. [35], SME and SSE have comparable HP abilities throughout the short and long terms. In contrast to SME, the SSE showed a notable reduction in HP rate over time. Systematic assessments as of late 2025 show that the SME sustains more stable rates of hearing preservation after a year. The body’s fibrotic reaction and ongoing pressure on fragile lateral wall structures are frequently blamed for the SSE’s degeneration. Although performance frequently levels out after 24 months, studies support the SME design for enhanced speech perception during the first six months and overall melody perception. For cochleae with anatomical differences or malformations where a pre-curved electrode would not fit properly, the SSE is still the recommended option. To maintain residual hearing following cochlear implantation, a variety of hearing preservation electrode arrays have been developed for patients with preoperative residual hearing [39]. Most of these electrodes are straight in design and lack a pre-curved or contoured configuration.

Cochlear dimensions are a critical determinant of successful cochlear implantation. Because the size and morphology of the cochlea differ substantially between individuals, these anatomical variations directly affect the ability of the electrode array to effectively stimulate the auditory nerve. The average cochlear duct length is approximately 30 mm, although considerable interindividual variability exists, influenced by factors such as age, genetic background, and sex [40]. An inappropriate match between electrode array length and cochlear size may result in several technical and functional problems. For instance, an electrode that is too long may be difficult to fully insert into a smaller cochlea, whereas a relatively short electrode may provide insufficient cochlear coverage and suboptimal neural stimulation [41]. Meticulous preoperative evaluation in cochlear implantation, including precise assessment of the cochlear duct length (CDL), plays a critical role in selecting an appropriately sized electrode array. A tablet-based planning application provides a user-friendly and efficient platform for visualizing temporal bone anatomy, assessing its spatial proportions, and calculating the CDL. Considerable variability in CDL was identified, with significantly greater measurements in male patients, whereas no meaningful differences were found with respect to laterality or age. In the majority of cases, the cochlear length exceeded 31.0 mm [40,42,43,44]. Because many patients using electro-acoustic stimulation (EAS) experience progressive loss of residual hearing as part of the natural progression of hearing impairment, extended cochlear coverage with longer electrode arrays can be advantageous by enabling more accurate place–pitch alignment. Beyond anticipating future auditory decline, the use of longer electrodes in EAS systems also allows greater flexibility in programming options, supporting a wider range of mapping strategies to optimize auditory performance [40].

In recent years, however, increasing attention has been directed toward the risk of electrode migration, a clinically relevant issue because it may necessitate revision surgery for electrode repositioning or adjustment. Electrode migration following cochlear implantation appears to occur more frequently than has been traditionally recognized. Electrode migration is an infrequent complication of cochlear implantation; however, it represents the second most common indication for revision surgery requiring electrode repositioning. Previous reports indicate that electrode migration accounts for approximately 1–15% of all cochlear implant revision procedures [44]. Despite these observations, the pathophysiological mechanisms underlying electrode migration remain incompletely understood. In pediatric patients, electrode migration appears to occur more frequently, likely due to ongoing skull growth and progressive new bone formation within the mastoid cavity, which may contribute to electrode extrusion. In contrast, in adult patients, factors such as head trauma as well as intracochlear fibrosis or ossification have been proposed as potential contributors, facilitating gradual withdrawal of the electrode array from the cochlea. Accordingly, meticulous attention to secure electrode array fixation during surgery is essential. These findings emphasize the critical role of postoperative imaging in the assessment of cochlear implant positioning.

## 5. Limitations

This review is limited by heterogeneity in study designs, electrode models, outcome measures, and follow-up durations. Randomized controlled trials remain scarce, and residual confounding cannot be excluded.

## 6. Conclusions

Based on evidence published between 2015 and 2025 and synthesized from 32 included studies, both slim straight (lateral wall) and slim perimodiolar electrode arrays achieve comparable overall speech perception outcomes in most adult and pediatric cohorts. While selected studies reported earlier or slightly higher CNC performance with perimodiolar arrays at specific time points, the majority of comparative data indicate that postoperative speech results are strongly influenced by intrascalar position and atraumatic scala tympani placement, rather than electrode geometry alone. Straight (lateral wall) arrays consistently demonstrated lower rates of scalar translocation and tip fold-over, and, in many studies, were associated with more reliable residual hearing preservation, particularly in electro-acoustic stimulation candidates. Although modern slim perimodiolar designs have reduced historical complication rates and show improved short-term hearing preservation compared with earlier pre-curved models, imaging-based studies still report a relatively higher vulnerability to intracochlear deviation when malposition occurs. Perimodiolar arrays may offer electrophysiological efficiency advantages due to closer modiolar proximity and lower stimulation thresholds; however, these theoretical benefits do not uniformly translate into superior long-term functional outcomes.

Overall, contemporary evidence supports the view that surgical technique, scala tympani integrity, individualized cochlear coverage, and secure electrode fixation are more critical determinants of success than electrode design alone. Electrode selection should therefore be individualized, taking into account cochlear anatomy, cochlear duct length, hearing preservation goals, and long-term programming flexibility, rather than relying solely on electrode configuration.

## Figures and Tables

**Table 1:** Risk of bias was assessed using ROBINS-I for non-randomized studies and RoB 2 for randomized trials. ROBINS-I Risk of Bias Assessment.

ROBINS-I Domain	Risk Level
Bias due to confounding	Moderate
Bias in selection of participants	Low
Bias in classification of interventions	Low
Bias due to deviations from intended interventions	Low
Bias due to missing data	Moderate
Bias in measurement of outcomes	Low
Bias in selection of reported results	Moderate
Overall risk of bias	Moderate

**Table 2:** The PRISMA search process identified 1284 records, of which 32 studies met the inclusion criteria after screening and eligibility assessment. (LW: lateral wall, PM: perimodiolar).

No.	First Author	Year	Journal	Study Design	Electrode Type Compared	Ref No.
1	Dhanasingh	2017	Hearing Research	Narrative review	LW vs. PM	[1]
2	O’Connell	2016	Otology & Neurotology	Retrospective cohort	CI522 vs. CI512	[2]
3	Jwair	2021	Laryngoscope	Meta-analysis	LW vs. PM	[3]
4	Ketterer	2022	Eur Arch Otorhinolaryngol	Retrospective cohort	LW vs. PM	[4]
5	Shaul	2018	J Laryngol Otol	Retrospective cohort	PM only	[5]
6	Ramos de Miguel	2022	J Clin Med	Temporal bone study	Slim PM	[6]
7	Giardina	2019	Ear Hear	Prospective cohort	Flexible LW	[7]
8	Zimmermann	2025	J Otolaryngol Head Neck Surg	Systematic review	LW vs. PM	[8]
9	MacPhail	2022	Otolaryngol Head Neck Surg	Retrospective cohort	Slim straight vs. slim modiolar	[9]
10	Pennington-FitzGerald	2025	Otolaryngol Head Neck Surg	Retrospective cohort	LW vs. PM	[10]
11	Fries	2025	Front Neurol	Retrospective cohort	LW vs. PM	[11]
12	Almuhawas	2025	Eur Arch Otorhinolaryngol	Prospective cohort	PM	[12]
13	Garaycochea	2020	Eur Arch Otorhinolaryngol	Comparative cohort	2 PM vs. 1 LW	[13]
14	O’Connell	2016	Laryngoscope Investig Otolaryngol	Review	LW vs. PM	[14]
15	Mewes	2020	Otology & Neurotology	Comparative cohort	PM designs	[15]
16	Zarowski	2020	Eur Arch Otorhinolaryngol	Pediatric cohort	LW vs. PM	[16]
17	Wanna	2015	Otology & Neurotology	Prospective cohort	LW vs. PM	[17]
18	Helbig	2015	Otology & Neurotology	Prospective cohort	Flexible LW	[18]
19	Shew	2021	Otology & Neurotology	Retrospective cohort	Slim modiolar	[19]
20	Hallin	2024	Acta Otolaryngologica	Retrospective cohort	LW vs. PM	[20]
21	Woodson	2020	Otology & Neurotology	Comparative cohort	Slim PM vs. slim LW	[21]
22	Liebscher	2021	Z Med Phys	Retrospective cohort	PM	[22]
23	Patro	2024	Otology & Neurotology	Comparative cohort	Mid-scala vs. LW	[23]
24	Varghese	2024	Otolaryngol Head Neck Surg	Retrospective cohort	Slim PM	[24]
25	Savoca	2023	Otolaryngol Head Neck Surg	Retrospective cohort	PM	[25]
26	Chabuz	2025	Laryngoscope	Cadaver study	PM	[26]
27	Zuniga	2017	Otology & Neurotology	Case series	PM	[27]
28	Dhanasingh	2024	Front Neurol	Narrative review	CI electrode designs	[28]
29	Gabrielpillai	2018	Otology & Neurotology	Retrospective cohort	Slim PM	[29]
30	von Mitzlaff	2021	Cochlear Implants Int	Retrospective cohort	LW	[30]
31	Magos	2024	Cochlear Implants Int	Retrospective cohort	LW vs. PM	[31]
32	Ha	2024	Eur Arch Otorhinolaryngol	Experimental study	Fixation techniques	[32]

## Data Availability

No new data were created or analyzed in this study.
